# Disseminated Tuberculosis With Myocarditis and Intracardiac Thrombus in a Previously Young Healthy Woman

**DOI:** 10.1016/j.jaccas.2021.08.020

**Published:** 2021-11-03

**Authors:** Mohd Asyiq Raffali, Syawal Faizal Muhammad, Patrick Tiau Wei Jyung, Diyana Farouk, Awatif Zohdi, Hamat Hamdi Che Hassan

**Affiliations:** Department of Medicine, University Kebangsaan Malaysia (UKM) Medical Centre, Kuala Lumpur, Malaysia

**Keywords:** cardiomyopathy, heart failure, myocarditis, thrombus, tuberculosis, ACE, angiotensin-converting enzyme, CT, computed tomography, ECG, electrocardiogram, EHRZ, ethambutol-isoniazid-rifampicin-pyrazinamide, LV, left ventricular, PCR, polymerase chain reaction, TB, tuberculosis

## Abstract

A 33-year-old woman with newly diagnosed disseminated tuberculosis presented with acute heart failure and incidental findings of intracardiac thrombus, demonstrating possible tuberculous myocarditis. (**Level of Difficulty: Intermediate.**)

## Central Illustration

**Figure:**
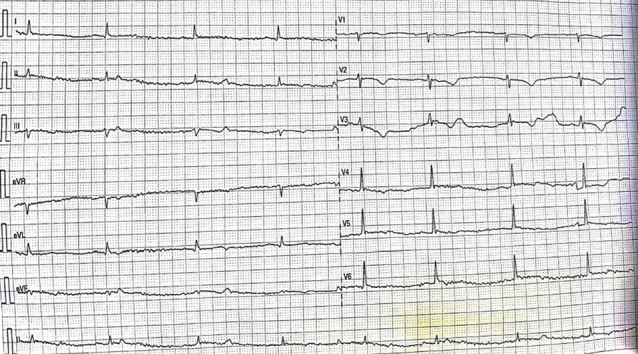

## History of Presentation

A 33-year-old woman presented to our emergency department as a result of syncope at work, preceded by fever and cough for 2 weeks, and loss of appetite and weight for 2 months. On arrival, she was tachypneic and short of breath. On examination, there were palpable cervical lymph nodes, generalized coarse crackles, and bilateral lower limb pitting edema. Her blood pressure was 100/40 mm Hg, with a heart rate of 110 beats/min, and her oxygen saturation was 92%, with a respiratory rate of 30 breaths/min.

**Learning Objectives**

• To understand the possibility of myocardial involvement in disseminated TB.
• To learn the management of tuberculous myocarditis and the challenges in its treatment.

## Past Medical History

She had a history of childhood bronchial asthma with no history of intensive care admission or endotracheal intubation.

## Differential Diagnosis

The differential diagnosis included viral or bacterial community-acquired pneumonia, acute decompensated heart failure, and pulmonary embolism. Multimodality imaging was required to make the diagnosis.

## Investigations

**Figure 1:**
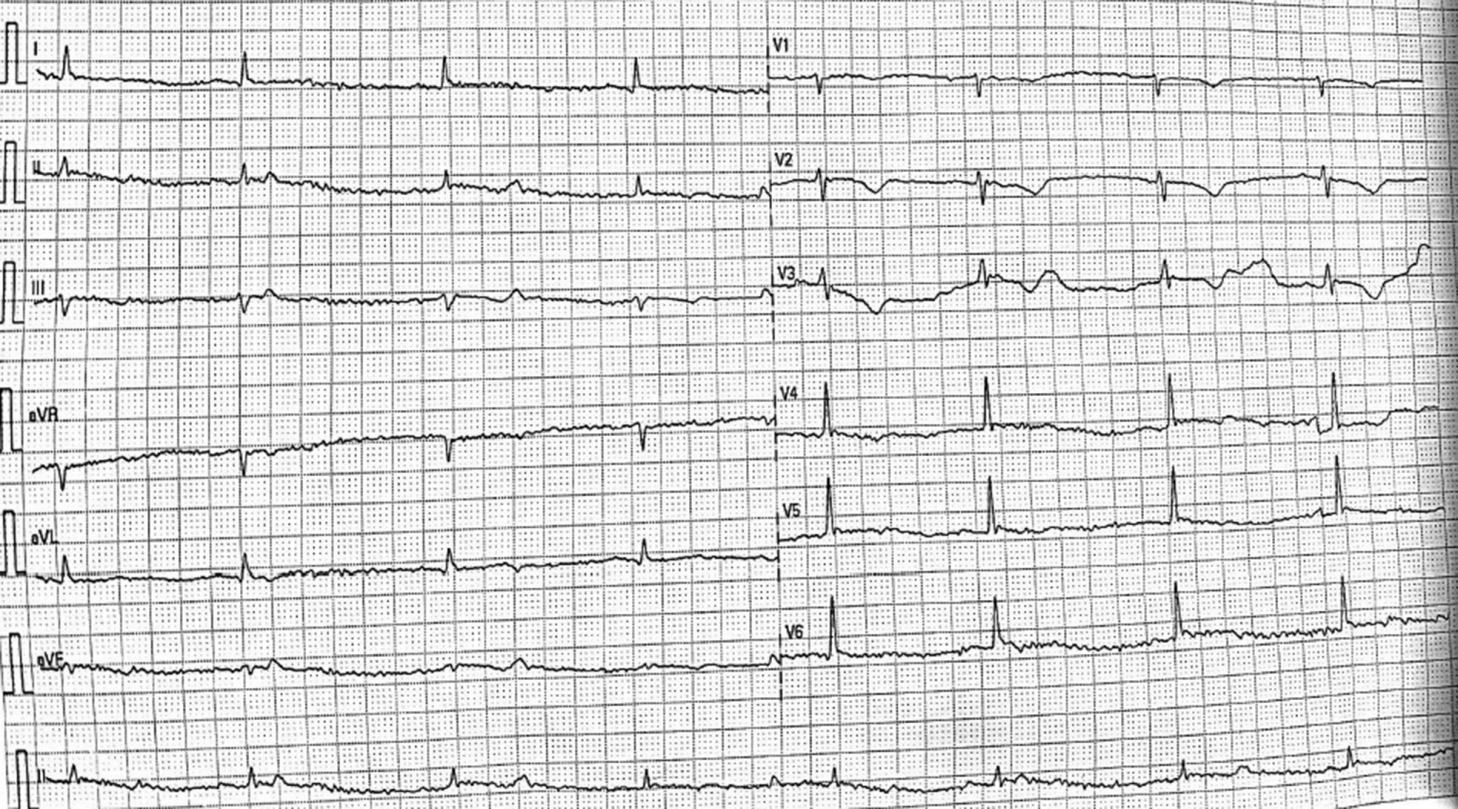
Electrocardiogram on Admission Showing Sinus Bradycardia With T-Wave Inversion in the Precordial Leads

**Figure 2:**
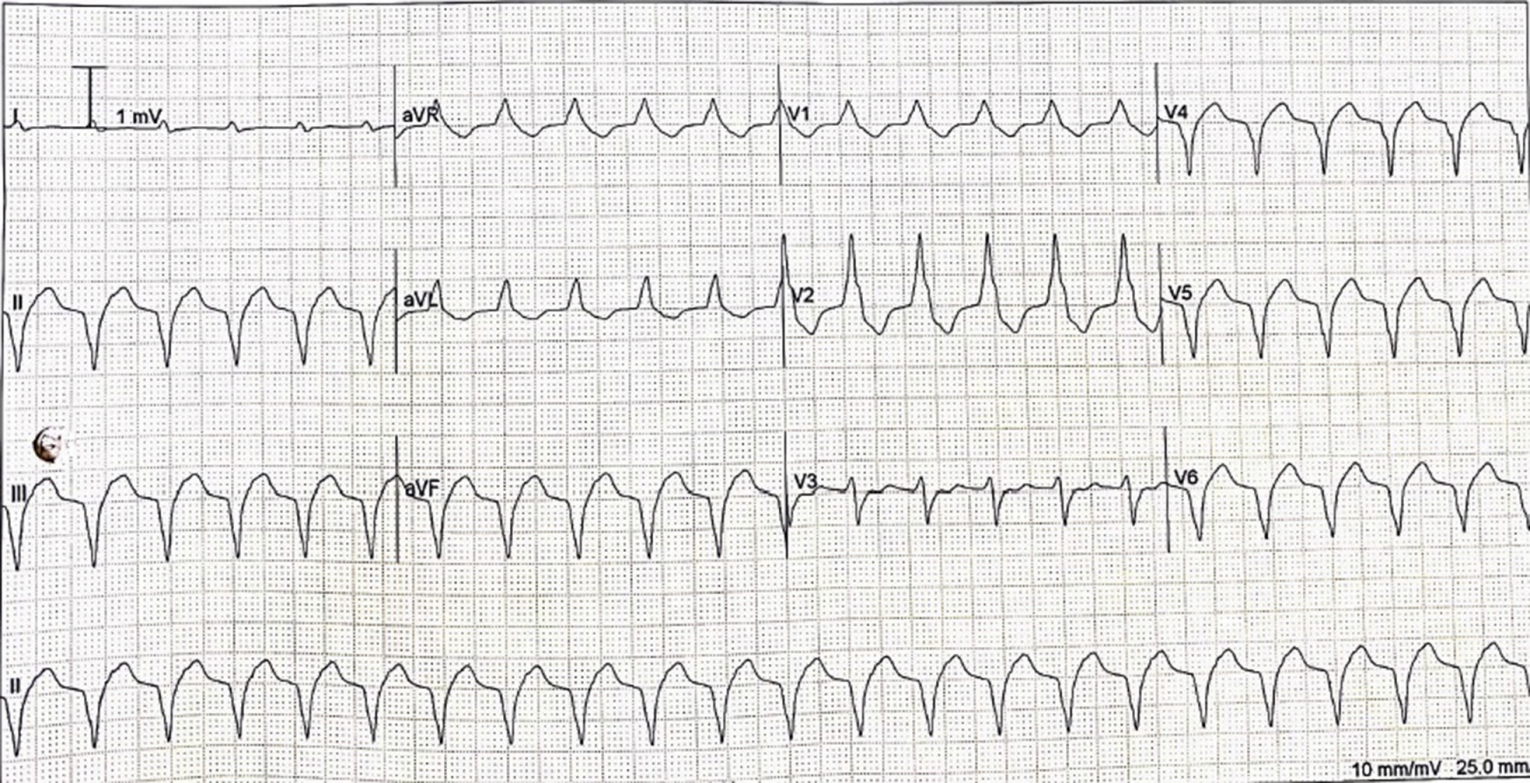
Electrocardiogram During Admission Showing Complete Right Bundle Branch Block

**Figure 3:**
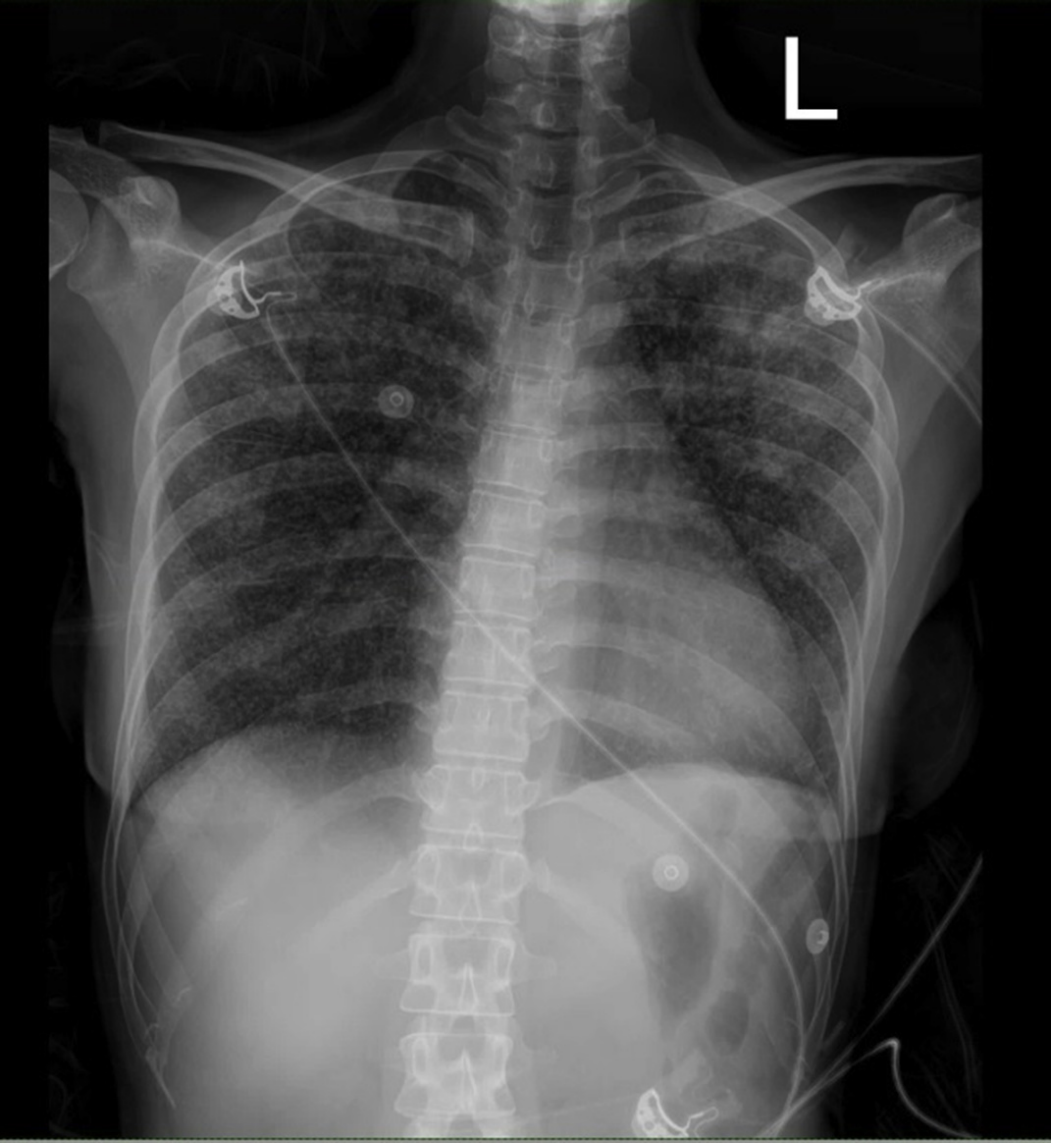
Chest Radiography Showing Diffuse Miliary Changes of the Lungs

**Figure 4:**
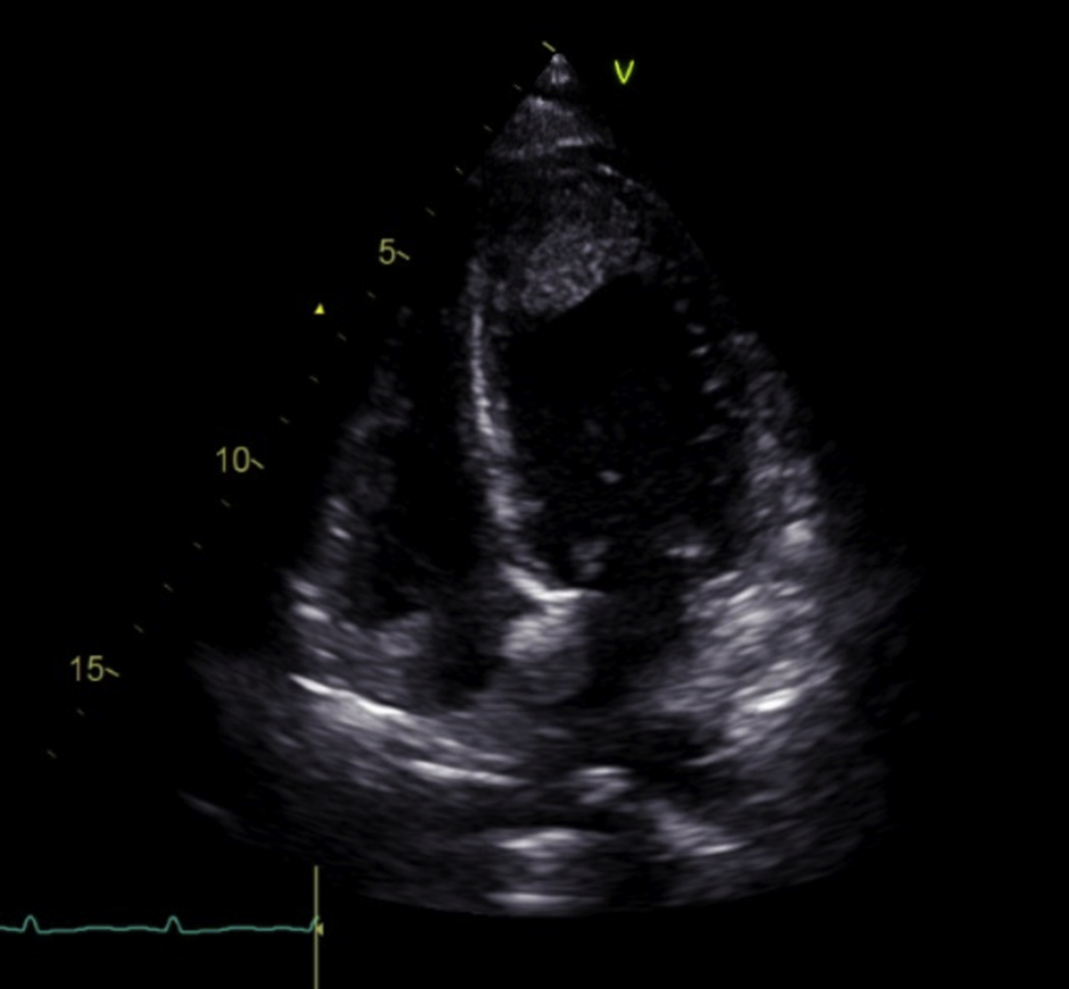
Still Image From Echocardiography on Admission Apical 4-chamber view showing global hypokinesia and apical thrombus.

**Figure 5:**
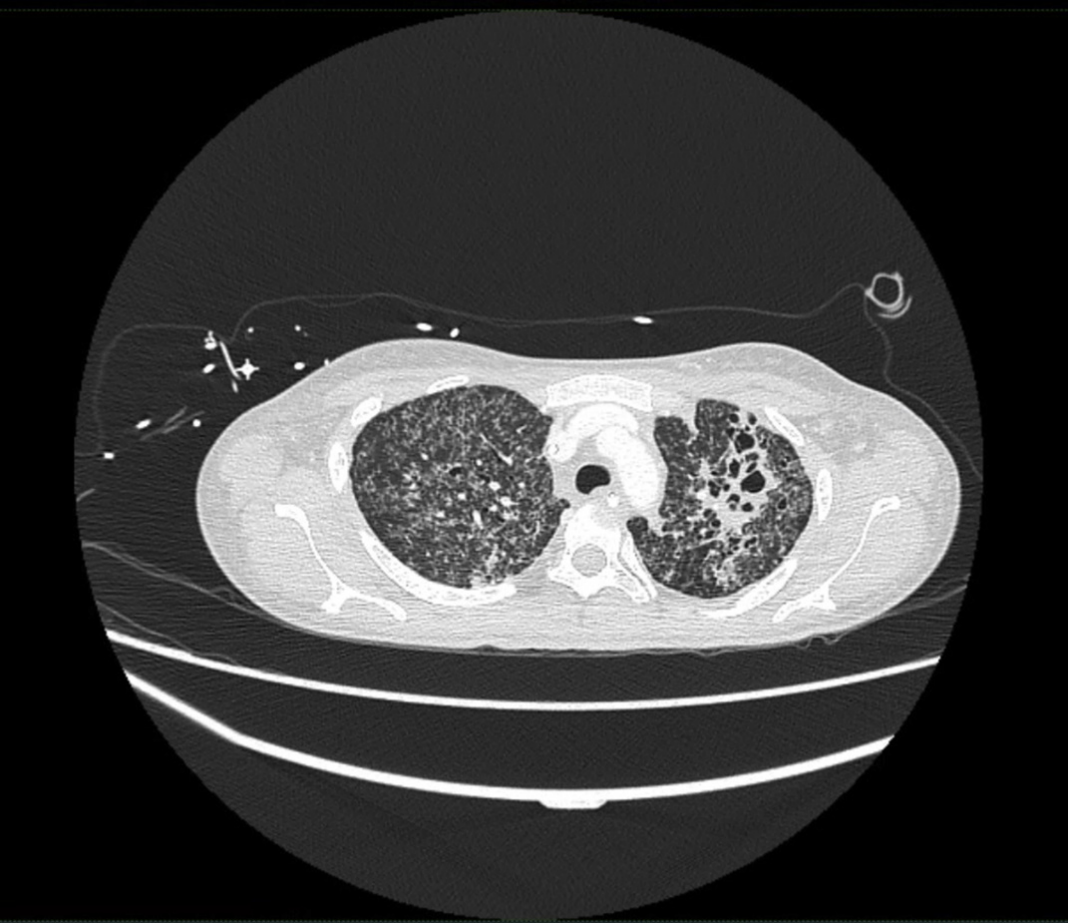
Chest Computed Tomography Showing Cavitation of the Upper Lobes of the Lungs

**Figure 6:**
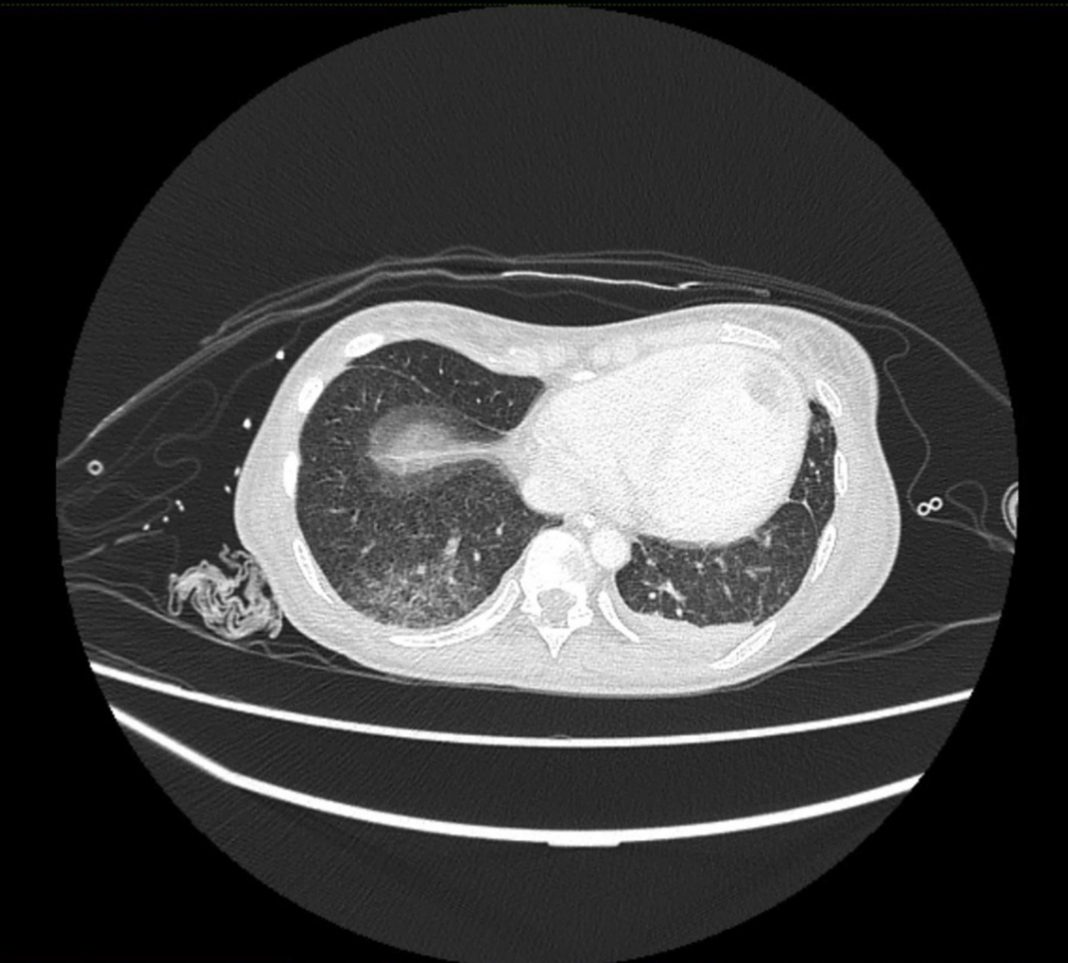
Chest Computed Tomography Showing Axial View of the Left Ventricular Apical Thrombus

**Figure 7:**
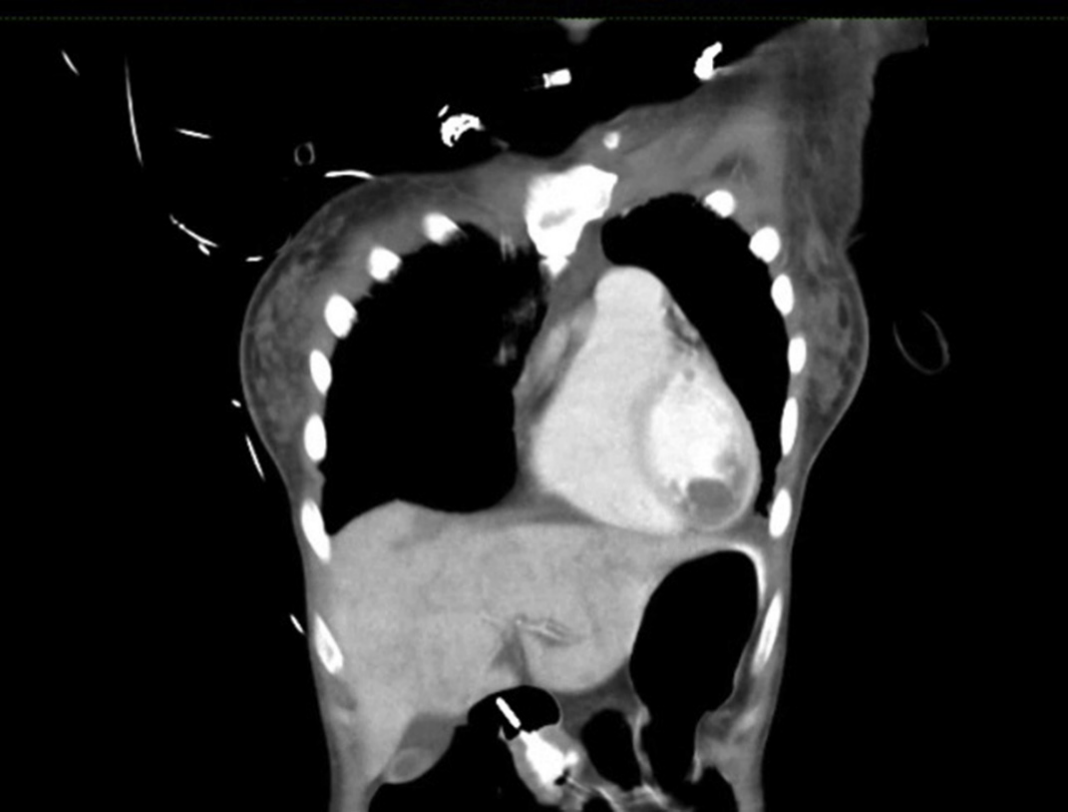
Chest Computed Tomography Showing Coronal View of the Left Ventricular Apical Thrombus

The electrocardiogram (ECG) showed sinus bradycardia with nonspecific T-wave inversion in the precordial leads, with intermittent complete right bundle branch block (Figures 1 and 2). Chest radiography showed diffused bilateral miliary changes (Figure 3). Echocardiography showed severely impaired global left ventricular (LV) systolic function with an estimated ejection fraction of 15%, the presence of an apical mural thrombus, and mild pericardial effusion. There were no features suggestive of myocardial infiltrative disease or other cardiac masses apart from the mural thrombus (Figure 4, Videos 1, 2, and 3). Blood biochemistry showed an elevated high-sensitivity troponin I level of 85 pg/mL and an elevated C-reactive protein level of 9.85 mg/dL. The result of a COVID-19 rapid molecular polymerase chain reaction was negative in this patient, along with HIV antibody. The patient was subsequently intubated for severe respiratory distress, and a tracheal aspirate showed the presence of numerous acid-fast bacilli. The patient subsequently required inotropic support with an intravenous norepinephrine infusion after her blood pressure dropped to 64/40 mm Hg after intubation. An empirical intravenous antibiotic, ceftriaxone, was also initiated to treat septic shock. Computed tomography (CT) of the thorax, abdomen, and pelvis showed disseminated TB involving the lungs, intestines, and bones, as well as a possible tuberculoma within the liver and spleen (Figure 5). CT also confirmed the presence of a mural thrombus within the left ventricle (Figures 6 and 7). Consequently, liver and renal impairment developed, with highest serum alanine transaminase of 1,183 U/L and creatinine of 146 μmol/L, likely in response to the hypotensive episode and sepsis.

## Management

Because the patient presented with disseminated TB, anti-TB antimicrobial agents were started immediately. The challenge was initiating anti-TB medication in a patient with liver and renal impairment secondary to mixed cardiogenic and septic shock. Bridging anti-TB medication was initially started, followed by the standard ethambutol-isoniazid-rifampicin-pyrazinamide (EHRZ) regimen on recovery of her liver function after 2 weeks. For the management of heart failure, an intravenous loop diuretic agent, furosemide, was started first, followed by initiation of a beta-blocker, oral bisoprolol, at 1.25 mg daily, as well as an angiotensin-converting enzyme (ACE) inhibitor, oral perindopril, at 2 mg daily. Anticoagulation with subcutaneous enoxaparin was started for the LV thrombus. Fortunately, no significant arrythmia developed in this patient throughout the admission, despite the intermittent right bundle branch block seen on the initial ECG.

## Discussion

Myocardial involvement in disseminated TB is rare in published reports, and it is usually discovered during postmortem examination (1). The prevalence of myocardial TB has been reported to range from 0.14% to 2% in various series, and it is often associated with pericarditis and pericardial effusion (2). As a result of depressed myocardial function, patients may present with ventricular arrythmias, congestive cardiac failure, and sudden cardiac arrest. Most case series show marked improvement in cardiac function on initiation of anti-TB antimicrobial agents. Therefore, prompt diagnosis of TB is vital for best patient outcomes. Cardiac magnetic resonance may aid in the diagnosis of TB myocarditis, by identifying features of tuberculous myocardial infiltration; however, given the overwhelming evidence of disseminated TB, the test was unwarranted in our patient. Other diagnostic testing such as endomyocardial biopsy could also help, but bear in mind the possibility of a nondiagnostic yield from the biopsy, as well as possible procedural complications (3,4).

## Follow-Up

**Figure 8:**
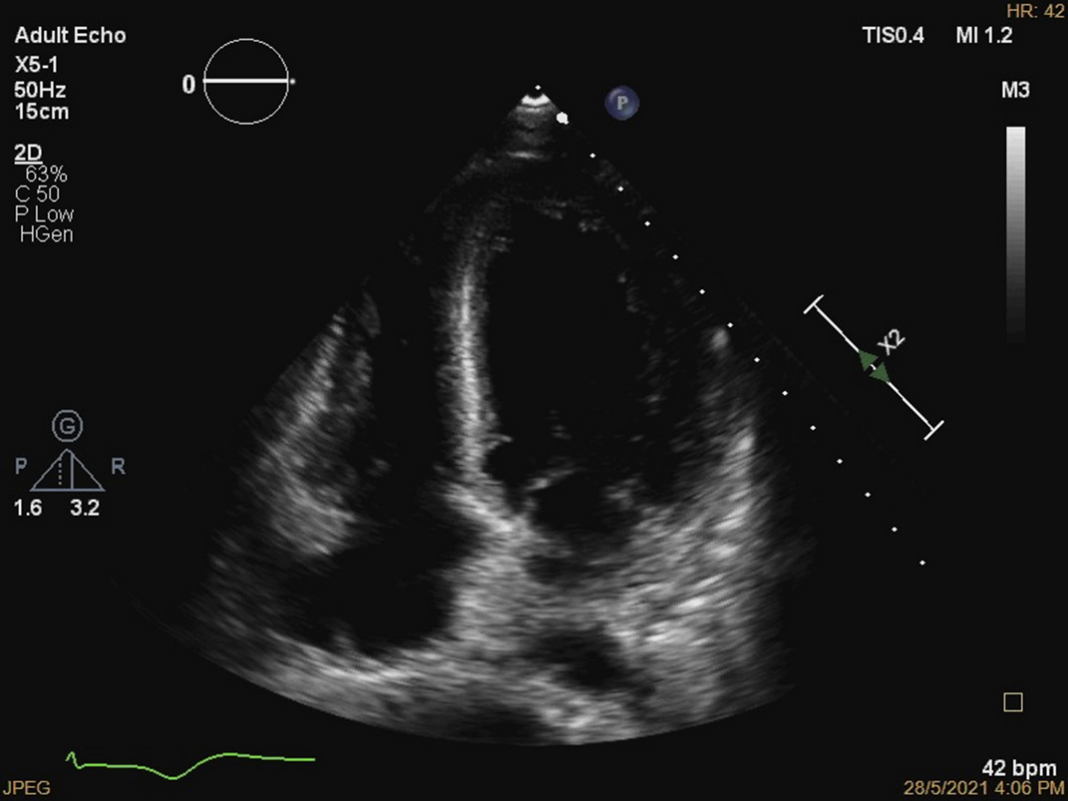
Still Image From Echocardiography Apical 4-chamber view after 3 weeks of antituberculosis treatment showing resolution of the left ventricular apical clot

Repeated echocardiography after 3 weeks of anti-TB medication and continuation of the oral β-blocker and ACE inhibitor showed an improved ejection fraction to 45% and nearly full resolution of the LV mural thrombus (Figure 8, Videos 4 and 5). There is a possibility that the thrombus could have consisted of TB bacilli in the form of a tuberculoma, such as described in a case report by Hajsadeghi et al (5), given its relatively rapid resolution. However, because there was no direct sampling of the clot, the question remains unanswered. Sputum acid-fast bacilli were absent afterward. The patient was discharged with an oral vitamin K antagonist, warfarin, after 3 weeks of subcutaneous enoxaparin treatment. Anticoagulation was planned for a minimum of 3 months with serial echocardiography to monitor LV function and thrombus resolution (6). For the treatment of disseminated TB, the plan was for the patient to undergo 2 months of intensive therapy with the EHRZ regimen and at least 7 months of maintenance with isoniazid and rifampicin.

## Conclusions

Despite being rarely encountered in a patient with disseminated TB, TB myocarditis should be considered in the differential diagnosis, especially in young patients. Timely anti-TB treatment aids in the resolution of the disease.

## Funding Support and Author Disclosures

The authors have reported that they have no relationships relevant to the contents of this paper to disclose.

## Appendix

Supplemental Video 1 Parasternal Long-Axis View of the Heart on Admission Showing Global Hypokinesia

Supplemental Video 2 Apical 4-Chamber View of the Heart on Admission Showing Large LV Apical Thrombus With Global Hypokinesia

Supplemental Video 3 Subcostal View of the Heart on Admission Showing Pericardial Effusion, LV Apical Thrombus, and Global Hypokinesia

Supplemental Video 4Apical 4-Chamber View of the Heart After 3 Weeks of Antituberculosis Treatment Showing Improved Contractility of the Heart and Resolution of the LV Apical Thrombus

Supplemental Video 5 Parasternal Long-Axis View of the Heart After 3 Weeks of Antituberculosis Treatment Showing Improved LV Contractility
